# Aplasia Cutis Congenita as a Sole Manifestation of Congenital Varicella Syndrome

**DOI:** 10.1155/2020/6147250

**Published:** 2020-07-21

**Authors:** Alexander K. C. Leung, Kin Fon Leong, Joseph M. Lam

**Affiliations:** ^1^Clinical Professor of Pediatrics, The University of Calgary and Pediatric Consultant, The Alberta Children's Hospital, Calgary, Alberta, T2M 0H5, Canada; ^2^Consultant Pediatric Dermatologist, The Pediatric Institute, Kuala Lumpur General Hospital, Kuala Lumpur, Malaysia; ^3^Department of Dermatology and Skin Sciences, University of British Columbia and Consultant Pediatric Dermatologist, The BC Children's Hospital Vancouver, Vancouver, British Columbia, V6H 3V4, Canada

## Abstract

Aplasia cutis congenita following maternal varicella is well known. On the other hand, aplasia cutis congenita as the sole manifestation of congenital varicella syndrome is very rare. A perusal of the literature revealed only one case. We report two infants with aplasia cutis congenita as the sole manifestation of congenital varicella syndrome.

## 1. Introduction

Fetal infection following maternal varicella, especially during the first 20 weeks of gestation, can result in varicella embryopathy, also known as congenital varicella syndrome or fetal varicella syndrome. This constellation of malformations was first described by LaForet and Lynch in 1947 [1]. We report a 5-day-old boy and a 17-day-old boy with aplasia cutis congenita as the sole manifestation of congenital varicella syndrome. A perusal of the literature revealed only one case [2], to which we are adding two more, to alert readers of such as an association.

## 2. Case Reports

### 2.1. Case 1

A 5-day-old Malay infant boy from Malaysia was born to a 28-year-old primigravid woman at 37 weeks gestation, following an uncomplicated normal spontaneous vaginal delivery. The Apgar score was 6 and 9 at 1 minute and 5 minutes, respectively. The birth weight was 2.6 kg, length was 48 cm, and head circumference was 34 cm. At birth, a linear skin defect was noted on the right forearm extending to the elbow. The neonatal course was otherwise uneventful.

**Figure 1:**
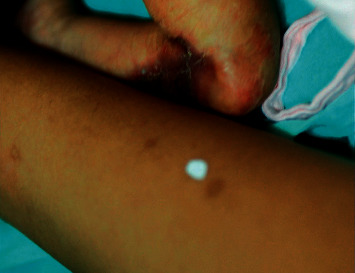
Mother (left) with hyperpigmented and hypopigmented scars on the left arm and infant (right) with aplasia cutis congenita on the right forearm.

At around the 16^th^ week of gestation, the mother experienced constitutional symptoms of malaise and fever. Two days later, she developed an itchy rash, which started as rose-colored macules, progressing rapidly to become papules, vesicles, pustules, and crusts. New lesions appeared in crops every one to two days, and lesions at different stages of development were seen. The distribution of the lesions was typically central, with the greatest concentrations on the trunk. After the rash had subsided, she was left with hyperpigmented and hypopigmented scars in lesional areas consistent with prior varicella infection (Figure 1). The mother did not recall having varicella or varicella vaccination in the past. A diagnosis of maternal varicella was made on clinical grounds. Parents were nonconsanguineous. There was no history of maternal medications taken during pregnancy.

**Figure 2:**
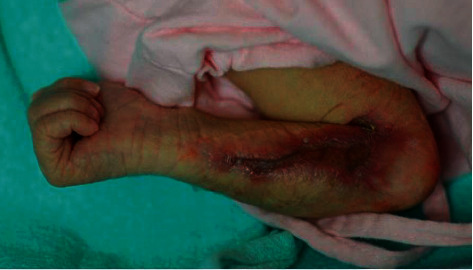
A 5-day-old infant with aplasia cutis congenita.

On physical examination, there was a linear skin defect involving two-thirds of the length of the right forearm extending to the elbow, along the distribution of the T1 dermatome (Figure 2). The lesion was depressed in comparison with the surrounding skin. There was a thin transparent membrane at the site of the skin defect, and the underlying subcutaneous structures were obvious to the naked eye. The skin surrounding the defect was erythematous and indurated. The rest of the physical examination was unremarkable. In particular, there were no dysmorphic features.

A clinical diagnosis of aplasia cutis congenita secondary to maternal varicella was made. The infant was seen by an ophthalmologist, a neurologist, and an orthopedic surgeon who did not detect any other anomalies. Cranial MRI was normal. The wound was cleaned daily with betadine solution. Fucidin acid cream was applied twice daily to the wound which was then covered with a nonadhesive sterile dressing. The wound healed in 34 days. The infant was referred to a plastic surgeon for reconstructive surgery. The parents were happy with the esthetic outcome. There was no functional impairment.

### 2.2. Case 2

A 17-day-old Malay infant boy from Malaysia was referred because of an extensive scar on the left flank, which was noted at birth. There was no history of vesiculobullous lesions. The infant was born to a gravida 2 para 1 30-year-old mother at term following an uncomplicated normal spontaneous vaginal delivery. The Apgar score was 7 and 10 at 1 minute and 5 minutes, respectively. The infant's birth weight was 2.7 kg, length was 47 cm, and head circumference was 35 cm. The infant was breast-fed and thriving. The neonatal course was unremarkable.

At around 15^th^ week of gestation, the mother developed an intensely pruritic rash consisting of erythematous macules, papules, pustules, and crusts, which appeared in crops. The rash was extensive, with the greatest concentration on the trunk. The lesions were typical of varicella. The mother was tested for varicella, and her serum varicella-zoster specific IgM was positive. She was treated with acyclovir 800 mg orally four times a day for five days. The maternal health was otherwise unremarkable. She was not on any other medications. There was no history of consanguinity and no family history of similar skin lesions.

**Figure 3:**
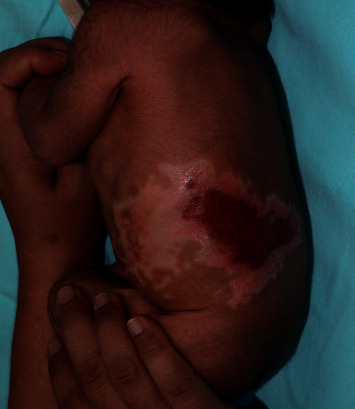
A 17-day-old infant with aplasia cutis congenita over the left flank, presenting as a whitish scar with erosion on the posterior aspect of the scar (side view).

**Figure 4:**
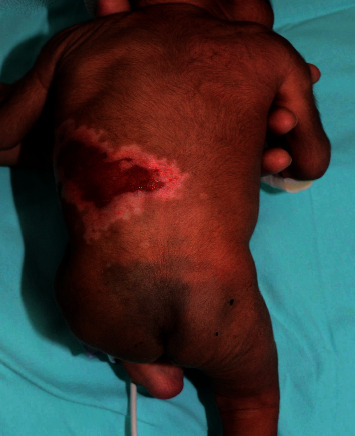
Same 17-day-old infant with aplasia cutis congenita over the left flank (back view).

On physical examination, the infant was alert and not in distress. Vital signs were normal. There was an extensive irregular, depressed, white scar over the left flank corresponding to the distribution of the T8 and T9 dermatomes (Figures 3 and 4). An area of erosion was noted on the posterior aspect of the scar. The rest of the physical examination was unremarkable.

A clinical diagnosis of aplasia cutis congenita secondary to maternal varicella was made. The infant's varicella-zoster specific IgM was negative. On the other hand, the varicella-zoster specific IgG was elevated at 3011 mIU/ml (>100 mIU/ml is considered as positive).

The infant was seen in consultation by various specialists, including a neurologist, an ophthalmologist, and an orthopedic surgeon, who could not detect other anomalies. He was referred to a plastic surgeon for follow-up care. The parents were happy with the esthetic outcome. There was no functional impairment.

## 3. Discussion

Humans are the only known reservoir for the varicella-zoster virus [3]. The virus is transmitted by direct contact with varicella or zoster lesions or by inhalation of infected airborne droplets [3]. The incidence of varicella has been estimated at 0.1–0.7 per 1000 pregnancies [4]. Up to 25% of the infants born to women who contract varicella may become infected [4, 5]. Approximately 2% of fetuses exposed to maternal varicella infection in the first 20 weeks (usually between 13 and 20 weeks) of pregnancy have features of congenital varicella syndrome [4, 6, 7].

Congenital varicella syndrome is characterized by a number of clinical manifestations: premature labor and small for gestational age; cutaneous lesions (e.g., scars in a dermatomal distribution and aplasia cutis congenita); CNS and peripheral nervous system abnormalities (e.g., microcephaly, hydrocephalus, cortical/cerebellar atrophy, mental retardation, facial nerve palsy, phrenic nerve palsy, recurrent laryngeal nerve palsy, bulbar palsy, brachial plexus palsy, and intracranial calcifications); ocular abnormalities (e.g., cataracts, nystagmus, microphthalmia, chorioretinitis, and optic atrophy); autonomic nervous system dysfunction (e.g., Horner syndrome, neurogenic bladder, dysphagia, and anal sphincter dysfunction); neuromuscular/orthopedic abnormalities (e.g., talipes equinovarus or calcaneovalgus deformity, hypoplasia/atrophy of the limb, rudimentary digit, hypoplasia of ribs, and scoliosis); gastrointestinal anomalies (e.g., duodenal stenosis, jejunoileal atresia, Meckel diverticulum, colonic atresia, colonic stricture, small left colon syndrome, and sigmoid atresia); and genitourinary anomalies (e.g., hydronephrosis, hydroureter, renal dysplasia, renal agenesis, and undescended testes) [6–18].

In the first case, varicella infection in the mother was diagnosed on clinical grounds following a classic presentation of varicella and the typical residual scars. Leung et al. examined 986 randomly selected children (519 boys, 467 girls) who had varicella at least one year previously for the presence of scars resulting from varicella [19]. The authors found that 96 (18.5%) boys and 88 (18.8%) girls had varicella scars, giving rise to an overall prevalence of 18.7%. The mean number of scars in the 184 children was 2.8 (standard deviation 1.9). The scars were hypopigmented in 160, hyperpigmented in 32, hypertrophic in 32, and depressed in 38 children. Two children had keloids. As varicella tends to be more severe in adults than in children, it is conceivable that adults have more scars from varicella infection. In the first case, the diagnosis of congenital varicella syndrome is based on evidence of maternal varicella infection during pregnancy and presence of aplasia cutis congenita in the infant.

In the second case, the diagnosis of maternal varicella was based on typical clinical features of varicella, positive varicella-zoster specific IgM in the mother, as well as a positive varicella-zoster specific IgG in the infant. In one study, only 4 of 16 (25%) infants with clinical manifestations of intrauterine varicella infection had positive varicella-zoster specific IgM [20].

Aplasia cutis congenita or congenital absence of the skin is characterized by a localized or widespread, complete or partial absence of different layers of the skin at birth [21]. The condition can present at birth with lesions that have already healed with scarring or with a glistening absence of skin that heals with scarring, as is illustrated in our two cases. Frieden classified aplasia cutis congenita into 9 groups, depending on the site of the skin defect, associated anomalies, associated syndromes of malformation, and exposure to teratogens as causative agents [21]. Group 8 refers to aplasia cutis congenita linked to teratogens such as medications (e.g., methimazole) and intrauterine infections (e.g., varicella and herpes simplex) as is illustrated in the present cases. Aplasia cutis congenita as one of features of congenital varicella syndrome is well known. Srabstein et al. reported a newborn infant with aplasia cutis congenita on the left lower leg with flexion contracture at the ankle, knee, and hips, cutaneous scars in the lumbar area, bilateral cataracts, microphthalmia, and micrognathia [22]. The infant was born to a mother who had contracted varicella between 13 and 15 weeks' gestation. On the other hand, aplasia cutis congenita as a sole manifestation of congenital varicella syndrome is extremely rare. A perusal of the literature revealed only one case. Bailie reported a newborn infant who had congenital absence of the skin over the left side of the neck and shoulder [2]. There were no associated anomalies. The mother of this infant had varicella at 15 weeks' gestation. Our patients add two more examples of this association. In our opinion, aplasia cutis congenita as a sole manifestation of congenital varicella syndrome, though rare, is more common that it is generally appreciated. Recognizing aplasia cutis congenita as the sole manifestation of congenital varicella syndrome is important so that this association will not be overlooked.

## 4. Conclusion

Congenital varicella syndrome is rare. Information on congenital varicella syndrome is mainly obtained from case reports. The diagnosis is much easier if there is a clear history of maternal varicella infection during pregnancy and if the affected infant has a constellation of features of congenital varicella syndrome. The diagnosis may not be obvious if aplasia cutis congenita is the sole manifestation and if the maternal history of varicella is overlooked. The present two cases help to alert readers that aplasia cutis congenita can be the sole manifestation of congenital varicella syndrome.
